# The Wnt7b/β-catenin signaling pathway is involved in the protective action of calcitonin gene-related peptide on hyperoxia-induced lung injury in premature rats

**DOI:** 10.1186/s11658-018-0071-7

**Published:** 2018-01-25

**Authors:** Shaohua Wang, Hongxing Dang, Feng Xu, Jian Deng, Xuemei Zheng

**Affiliations:** 1 Neonatal Intensive Care Unit, Women and Children Health Institute of Futian, University of South China, Jintian South Road No. 2002, Futian district, Shen Zhen, 518045 China; 2 Pediatric Intensive Care Unit, Children’s Hospital of Chongqing Medical University, Ministry of Education Key Laboratory of Child Development and Disorders, Yu Zhong, Chongqing, 400014 China

**Keywords:** CGRP, Lung injury, Wnt7b, β-catenin

## Abstract

**Background:**

Calcitonin gene-related peptide (CGRP) can protect against hyperoxia-induced lung injury, making the upregulation of CGRP a potential therapeutic approach for this type of injury. However, the effects of CGRP on the Wnt7b/β-catenin signaling pathway are unclear. In this study, we investigated the roles of CGRP and the Wnt7b/β-catenin signaling pathway in hyperoxia-induced lung injury.

**Methods:**

Premature Sprague Dawley (SD) rats were exposed to 21, 40, 60 and 95% oxygen for 3, 7 and 14 days. The animals’ body weights, survival rates and endogenous CGRP levels were measured. Lung samples were harvested for histological analyses and measurements of malondialdehyde (MDA) concentration and total antioxidant capacity (TAOC). We also assessed the MDA concentration and TAOC in the lung tissues after administration of 200 nmol/kg CGRP_8–37_ (a CGRP antagonist). Finally, alveolar epithelial type II (AEC II) cells were isolated from premature rats, exposed to 21 or 95% oxygen for 3, 7 and 14 days, and treated with 10^− 8^ mol/l exogenous CGRP. The protein expressions of Wnt7b and β-catenin were assessed using western blotting, and TCF and c-myc mRNA expressions were assessed using qPCR.

**Results:**

Rats exposed to 60 and 95% oxygen had significantly lower body weights and survival rates than the 21 and 40% groups, and the decrease was time dependent. Endogenous CGRP was elevated in the lung tissues of premature rats exposed to 95% oxygen. CGRP_8–37_ induced apparent inflammation in the lung tissue and alveolar structural remodeling. In addition, the expression levels of Wnt7b and β-catenin were markedly increased after exposure for 3 days. They peaked at 7 days, then declined at 14 days. The levels of TCF/c-myc in AEC II cells increased significantly after CGRP treatment when compared with cells that had only undergone hyperoxia.

**Conclusions:**

CGRP protected against hyperoxia-induced lung injury in premature rats. This process involves the Wnt7b/β-catenin signaling pathway.

## Background

Bronchopulmonary dysplasia (BPD) is characterized by oxidative injury and developmental stagnation of immature lung tissue caused by mechanical ventilation and long-term hyperoxia [1–4]. There is no effective treatment, meaning that BPD is still a major threat to pre-term infants, especially those with an extremely low birth weight. Understanding the effects of hyperoxia on the development of the immature lung and elucidating the underlying mechanisms is critically important.

Recent studies have shown that lung development and injury are complicated processes that involve numerous cytokines and signaling pathways [5, 6]. Calcitonin gene-related peptide (CGRP) is one of the sensory neuropeptides secreted by sensory nerve C-fiber terminals in the respiratory tract and neuroendocrine cells [7, 8]. It has dual functions of being both an extracellular signaling molecule and a cell regulatory factor involved in cell proliferation and differentiation, embryonic lung development, and respiratory self-repair processes [9].

Previous studies indicated that CGRP has a protective role against hyperoxia-induced DNA damage and lung injury in type II alveolar epithelial (AEC II) cells and neonatal rats [10, 11]. Another previous report demonstrated that CGRP is associated with the regulation of inflammatory cytokines [12]. However, the signaling pathways underlying the protective effects of CGRP on hyperoxia-induced lung injury have yet to be elucidated.

The protective effect of CGRP in AEC II cells under hyperoxia has been reported to involve the and Wnt 7b/β-catenin pathway [13]. The Wnt pathway is a highly conserved signal transduction pathway that plays an important role in the pathogenesis of several development-related lung diseases [14–16]. The Wnt family includes 19 glycoproteins encoded by the Wnt gene [17]. The expressions of Wnt2, Wnt2/Wnt13, Wnt5a, Wnt7b and Wnt11 have been detected in embryonic lung tissue [18, 19]. A high level of Wnt7b expression has been reported in developmental airway epithelial cells, and inactivation of Wnt7b leads to perinatal death of mice due to lung developmental defects, such as inhibited proliferation of epithelial and stromal cells and bronchial branching disorders [20, 21]. In addition, β-catenin, an important component of the Wnt signaling pathway, has been detected in the cytoplasm and nuclei of original and differentiated alveolar epithelial cells, suggesting its involvement in the development of terminal airways and alveoli [18].

This study was designed to elucidate the role of CGRP and the Wnt 7b/β-catenin pathway in hyperoxia-induced lung injury. We established a hyperoxia-induced lung injury model in premature Sprague Dawley (SD) rats, evaluated the dynamic changes in CGRP expression in the lung tissues, and measured radial alveolar counts (RAC), malondialdehyde (MDA) concentration and total antioxidant capacity (TOAC) in a rat model exposed to CGRP_8–37_ (a CGRP antagonist). The expression levels of a number of key components in the Wnt pathway, including Wnt7b, β-catenin, TCF and c-myc, were also assessed in AEC II isolated from premature SD rats.

## Methods

### Animal experiments

Specific pathogen-free (SPF) SD rats (aged 8 weeks, weighing 180–200 g) were purchased from the Experimental Animal Center of Chongqing Medical University. The animal study was approved by the Animal Ethical Committee of Chongqing Medical University (No. 20120107563). Male and female rats were housed in the same cage at a 1:1 ratio for mating. On the second day, female rats were examined for vaginal plugs. The day on which a vaginal plug was first observed was designated as Day 0 of pregnancy. On day 22 of pregnancy, baby rats were obtained by cesarean section for follow-up experiments.

We performed two series of experiments. In the first, surviving premature rats were randomly divided into four groups with 18 animals per group. The four groups of rats were raised with regular air (21% oxygen) or 40%, 60% or 95% oxygen for 3, 7 and 14 days. The four groups were respectively named 21%, 40%, 60% and 95%. At the specified times, body weight, survival rate and oxidative stress indicators including RAC, MDA and TAOC were evaluated.

CGRP_8–37_, as a specific CGRP antagonist, was used to inhibit the activity of CGRP in the second series of experiments, with 200 nmol/kg CGRP_8–37_ having been found as the right dosage to inhibit the activity of CGRP. In this second series, premature rats were again randomly divided into four groups with 18 animals per group. The control rats were raised with regular air (21%) oxygen. The other three groups were raised with 95% oxygen. Two of these groups of rats received an intraperitoneal injection of sterile normal saline (NS) or CGRP_8–37_ (200 nmol/kg). The four groups were named 21%, 95%, 95% + CGRP_8–37_ and 95% + NS. Each animal was weighed on days 0, 3, 7, and 14, and the survival rate was documented. The lung histology, oxidative stress indicators, including RAC value, MDA and TAOC, and endogenous CGRP level were also evaluated on days 3, 7 and 14.

### Cell experiments

Lungs from premature baby rats were cut into small pieces (~ 1 mm^3^) in precooled PBS. AEC II cells were obtained via trypsin digestion, followed by repeated adherence and centrifugation to remove fibroblasts. The AEC II cells were cultured in Dulbecco’s modified Eagle’s medium (DMEM) containing 10% FBS (Gibco) and divided into four groups. All four groups were cultured with 5% CO_2_ at 37 °C. The differences in culture conditions were: 21% O_2_ (group name 21%); 95% O_2_ (group name 95%); 21% O_2_ with 10^− 8^ mol/l CGRP in the cell medium (group name 21% + CGRP); and 95% O_2_ with 10^− 8^ mol/l CGRP in the cell medium (group name 95% + CGRP). AEC II cells from each group were harvested for experiments.

### CGRP radioimmunoassay

Endogenous CGRP levels in the lung tissues were determined via radioimmunoassay as previously described [22]. Briefly, 100 μl of CGRP antibody (1:130,000 dilution) and 100 μl of ^125^I–CGRP_8–37_ were added to each sample and to the standards. The samples were incubated with radiolabeled peptide and antiserum for 24 h at 4 °C. Unbound tracer was removed by adding 500 μl of 0.1 M phosphate buffer (pH 7.4) containing 1% norite charcoal, 50 mM NaCl and 0.1% bovine serum albumin. This mixture was centrifuged at 4000 × g for 20 min at 4 °C, the supernatant was decanted, the radioactivity was measured with gamma scintillation spectrometry, and the amount of CGRP in the samples was estimated by comparing the percent bound radioactivity to a standard curve using a four-point nonlinear least squares regression analysis.

### Immunohistochemistry and radial alveolar count (RAC)

Lung morphology was assessed using hematoxylin and eosin (H&E) staining and the radial alveolar count (RAC). The lung tissues were fixed with 10% neutral-buffered formalin, embedded in paraffin, cut into 5-μm sections for 12-h H&E staining, and evaluated using light microscopy (Nikon, Japan) at 200× magnification.

The degree of alveolarization was quantified using the RAC and mean linear intercept methods, as previously described [23]. Briefly, a perpendicular line was drawn from the center of the respiratory bronchiole to the distal acinus. The mean linear intercept was measured by dividing the length of a line drawn across the lung section by the total number of septal wall intercepts encountered in 30 lines (40 μm apart) per lung image. Three measurements were obtained from each section (nine RACs/mean linear intercept per single rat).

### Measurement of MDA and TAOC

As an indicator of protein oxidation and lipid peroxidation, MDA was detected in homogenized lung tissues using the MDA assay kit (Nanjing Jiancheng Biological Engineering Research Institute). The results were recorded as nmol MDA/g of lung. The ferric reducing antioxidant power (FRAP) assay was applied to determine the TAOC of lung homogenates [19]. The FRAP reagent kit was provided by the Jiancheng Bioengineering Institute. The FRAP reagent included 30 mM acetate buffer (pH 3.6), 10 mM 2,4,6-tris(2-pyridyl)-1,3,5-triazine (TPTZ) solution, 20 mM FeCl_3_ solution and deionized water. The lung tissues were mixed with 1.5 ml of the FRAP reagent and incubated in the dark at room temperature for 15 min. The absorbance was measured at a wavelength of 593 nm, and the results were recorded as U/g of lung.

### Western blotting

Cells were lysed in lysis buffer, and the concentrations of total proteins were analyzed using a BCA Protein Assay kit (BioTeke Corporation). A total of 50 μg of protein for each sample was denatured in SDS-PAGE sample buffer and electrophoresed on a 10% SDS polyacrylamide gel (Boster). Resolved samples were then transferred onto a PVDF membrane by semi-dry blotting. The membrane was then blocked with TBS-Tween (TBST; 0.01 mol/l Tris-HCl, pH 7.5; 0.9% NaCl; 0.05% Tween 20) and 5% milk for 1 h at room temperature, and probed overnight at 4 °C with anti-rat Wnt7b rabbit (sc-32,865, 1:1000), anti-rat β-catenin rabbit (sc-65,480, 1:1000) and anti-rat phosphorylation β-catenin rabbit antibodies (sc-16,743, 1:1000; all from Santa Cruz Biotechnology). The first antibody solution was removed from the membrane, which was then washed 3 times with TBST, incubated for 1 h at room temperature with horseradish peroxidase-conjugated sheep anti-rabbit antibody (sc-2379, 1:1000; Santa Cruz Biotechnology), and washed 3 times with TBST. Chemiluminesecence was then detected on ECL films using the ChemiDocXRS image acquisition system to collect the images and Quantity One 4.5.0 software for analysis.

### Quantitative real-time PCR

Quantitative real-time PCR (qPCR) was performed for the transcription factor TCF and the target gene c-myc. RNA extraction was performed using the RNA TRIzol kit (Taraka) according to the manufacturer’s instructions. Template cDNA was obtained via reverse transcription of total RNA. The best-known denaturant is heat, so reverse transcription was carried out at 30 °C for 10 min, 42 °C for 30 min, 95 °C for 5 min and 4 °C for 5 min. The cDNA was immediately centrifuged at 4000 × g for 20 min. Synthetic template cDNA was stored at − 20 °C. The primer sequences for c-myc were 5’-GGAGAAACGAGCTGAAGCGTAG-3′ (forward primer), 5’-CAGCCAAGGTTGTGAGGTTAGG-3′ (reverse primer). The primer sequences for β-actin were 5’-TCACCCACACTGTGCCCATCTATGA-3′ (forward primer) and 5’-CATCGGAACCGCTCATTGCCGATAG-3′ (reverse primer). The primer sequences for TCF were 5’-ATGACACGGATGACGATGGG-3′ (forward primer) and 5’-GGACAGGTGGGACTGGTTGAG-3′ (reverse primer). The thermal cycling conditions were 3 min at 94 °C and 30 s for initial denaturation at 94 °C, followed by 30 cycles of 30 s at 55 °C and 1 min at 72 °C. Each experiment was performed in triplicate. The relative mRNA expression levels were determined using the 2^-△△Ct^ method [24].

### Statistical analyses

Statistical analyses were conducted using SPSS 19.0 software. Results are presented as the means ± standard deviation. Multiple groups were compared using variance analysis. Comparison between two groups was conducted using *t* tests. A *p* value of less than 0.05 was considered significant.

## Results

### Body weight and survival rate of premature rats

**Fig. 1:**
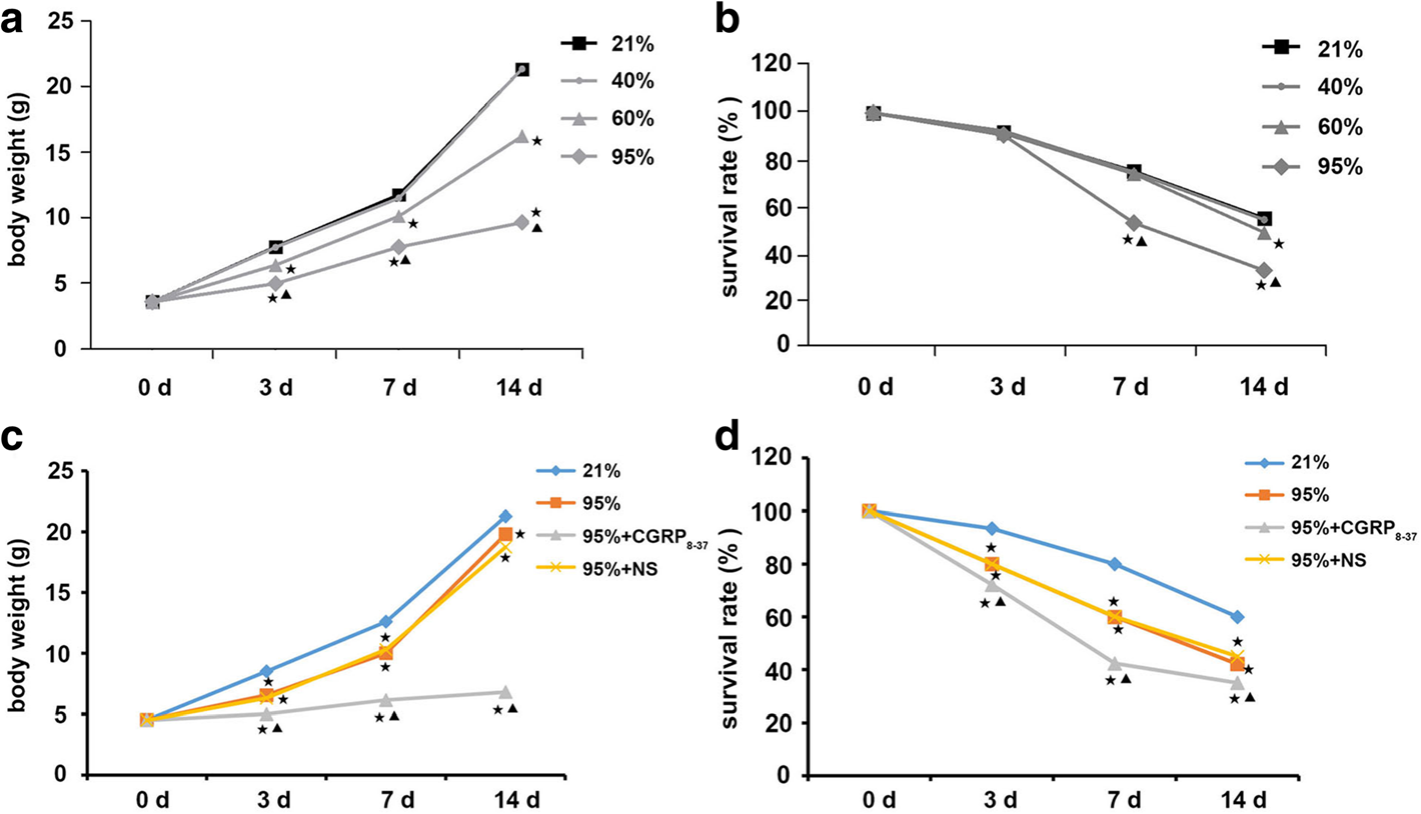
Body weight and survival rate of premature rats exposed to oxygen at different concentrations (21, 40, 60 or 95%) with or without exposure to CGRP_8–37_. **a** Changes in the body weight of premature rats exposed to 21, 40, 60 or 95% oxygen for 3, 7 and 14 days. **b** Changes in the survival rate of premature rats exposed to 21, 40, 60 or 95% oxygen for 3, 7 and 14 days. *p < 0.05 vs. 21% group; ^▲^p < 0.05 vs. 40% or 60% group. **c** Changes in the body weight of premature rats exposed to 21 or 95% oxygen with injection of CGRP_8–37_ or normal saline (NS). **d** Changes in the survival rate of premature rats exposed to 21 or 95% oxygen, with injection of CGRP_8–37_ or normal saline (NS). *p < 0.05 vs. 21% group; ^▲^p < 0.05 vs. 95% or 95% + NS group

The premature rats were exposed to 21, 40, 60 and 95% oxygen for 3, 7 and 14 days. The body weight and survival rate of premature rats exposed to 60 or 95% oxygen were significantly lower than those for the rats exposed to 21 and 40% oxygen (*p* < 0.05; Fig. 1a and b).

To understand the role of CGRP in oxidative injury of the premature lung, a CGRP antagonist, CGRP_8–37_, was used to block CGRP activity. After injection with CGRP_8–37_, the body weight and survival rate of premature rats exposed to 95% oxygen were significantly lower than those for the rats exposed to 95% oxygen alone (p < 0.05; Fig. 1c and d). These data suggest that CGRP_8–37_ injection caused a significant reduction in body weight with an increase in mortality rate.

### RAC value and MDA and TAOC levels in the lung tissues of premature rats

**Fig. 2:**
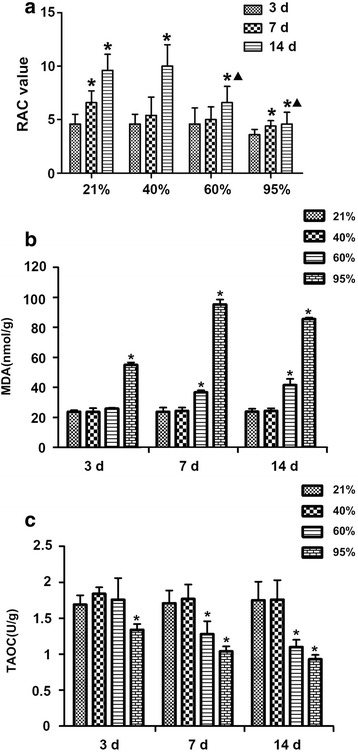
RAC value and MDA and TAOC levels in lung tissues of premature rats under hyperoxia. **a** RAC values in lung tissues of premature rats exposed to 21, 40, 60 or 95% oxygen (*n* = 6). **b** Changes in lung MDA levels (nmol/g) in premature rats exposed to 21, 40, 60 or 95% oxygen for 3, 7 and 14 days. **c** Changes in lung TAOC level (nmol/g) in premature rats exposed to 21, 40, 60 or 95% oxygen for 3, 7 and 14 days. *p < 0.05 vs. 3 days; ^▲^*p* < 0.05 vs. 21% group for the same period of exposure

The RAC values in premature rats exposed to 95% oxygen for 7 and 14 days were significantly lower than those determined for air control premature rats (Fig. 2a). The MDA content in the lung tissues of premature rats exposed to 60% oxygen for 7 and 14 days were significantly higher than those in premature rats exposed to 21% oxygen for 7 and 14 days. The lung tissue of premature rats exposed to 95% oxygen showed a gradual increase in MDA levels over time, and the levels were significantly higher than in premature rats exposed to 21% oxygen for the same time (Fig. 2b).

TAOC is an important factor for evaluating antioxidant capacity. The TAOC content in the lung tissues of premature rats exposed to 60% oxygen for 7 and 14 days was significantly lower than that in premature rats exposed to 21% oxygen for the same time. In addition, the TAOC content of the lung tissues of premature rats exposed to 95% oxygen for 3, 7, and 14 days was significantly lower than that of premature rats exposed to 21% oxygen for the same time (Fig. 2c). Therefore, 95% oxygen was used in the follow-up experiments.

**Fig. 3:**
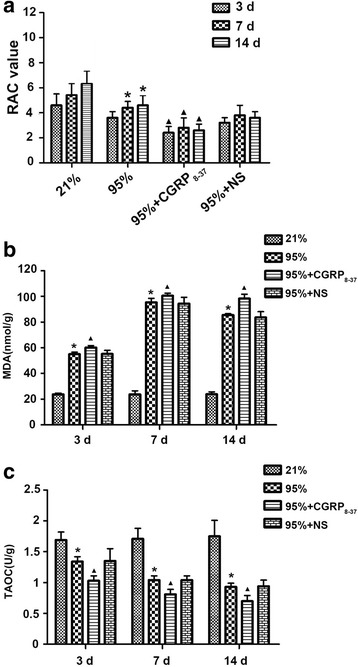
RAC value and MDA and TAOC levels after CGRP_8–37_ administration. **a** RAC values in lung tissues of premature rats (n = 6) exposed to 21 or 95% oxygen with or without administration of CGRP_8–37_ or normal saline (NS). **b** CGRP_8–37_ significantly increased MDA levels (nmol/g) in lung tissues exposed to 95% oxygen. **c** CGRP_8–37_ significantly reduced TAOC levels (U/g) in lung tissues exposed to 95% oxygen. *p < 0.05 vs. 21% group for the same period of exposure; ^▲^p < 0.05 vs. 95% group for the same period of exposure

CGRP_8–37_ exposure significantly reduced the RAC level in lung tissues exposed to 95% oxygen (Fig. 3a). The MDA and TAOC levels in lungs exposed to 95% oxygen for 3 days were respectively significantly higher and lower than in control rats exposed to 21% oxygen for 3 days. CGRP_8–37_ exposure significantly increased the TAOC level in lung tissues exposed to 95% oxygen for various periods (Fig. 3b and c), suggesting that it further abrogated the oxidant/antioxidant balance. These observations suggest that blocking CGRP activity significantly increased oxidative stress in premature rats.

### Lung histopathology and changes in endogenous CGRP

**Fig. 4:**
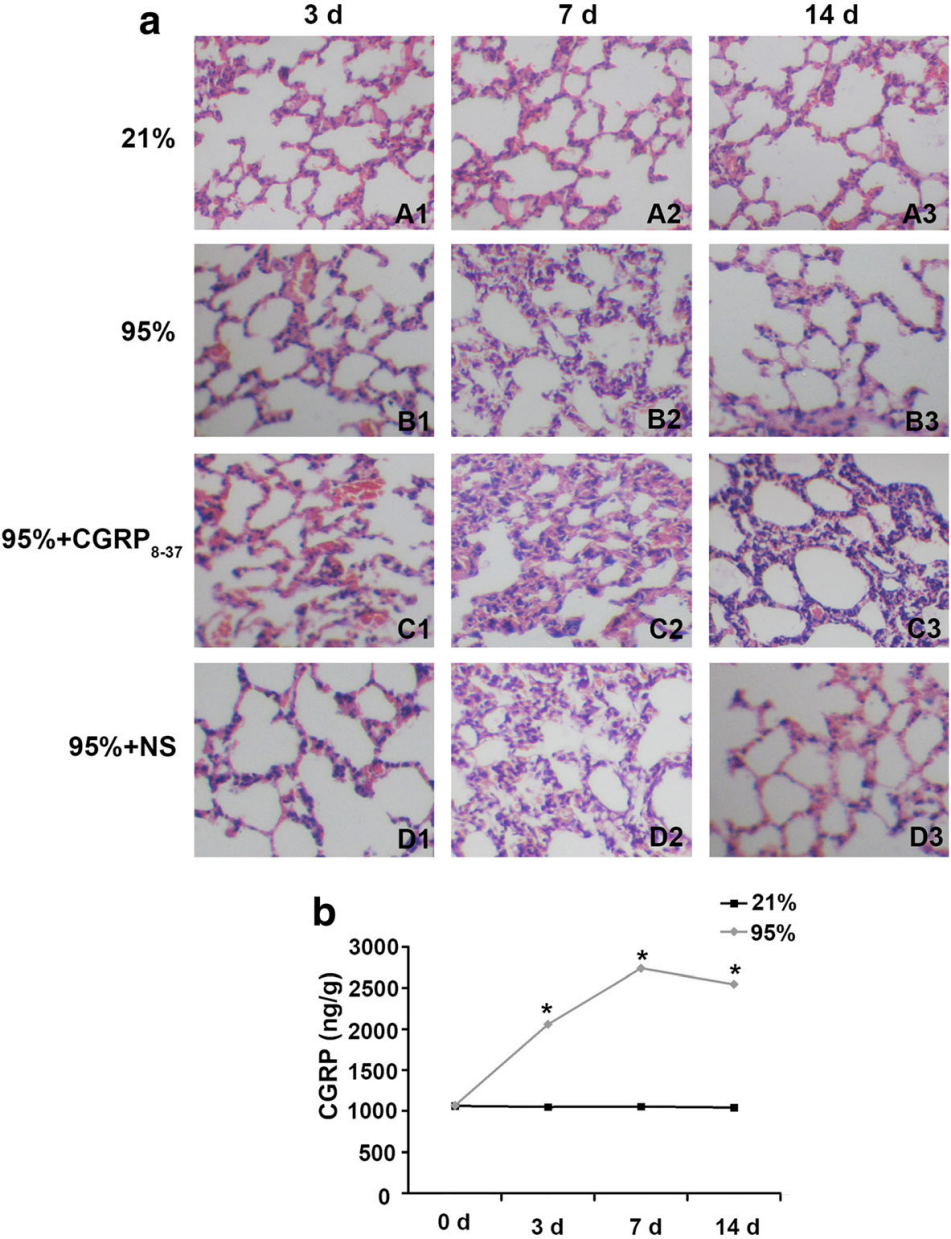
Lung histopathology and changes in endogenous CGRP levels. **a** Histopathological changes in the lung tissues of premature rats (n = 6) exposed to 21 or 95% oxygen with or without administration of CGRP_8–37_ or normal saline (NS). **b** Endogenous CGRP levels in lung tissues of premature rats exposed to 95% oxygen. *p < 0.05 vs. 21% group for the same period of exposure

We observed small blood vessel expansion and inflammatory cell infiltration in the lungs of premature rats exposed to 95% oxygen for 3 days (Fig. 4a). After 7 days of exposure to 95% oxygen, we observed a thickened alveolar wall and increased infiltration of the inflammatory cells in the lung tissues (Fig. 4a). After exposure to 95% oxygen for 14 days, reduced numbers of alveoli and thinning alveolar walls were observed (Fig. 4a). After exposure to CGRP_8–37_, we observed a more severe inflammatory response and alveolar structural remodeling in the lung tissue (Fig. 4a). No significant changes in the endogenous CGRP content were observed in the lung tissues of premature rats exposed to 21% oxygen for different periods of time. However, the endogenous CGRP levels in the lung tissues of premature rats exposed to 95% oxygen were significantly higher than those of premature rats exposed to 21% oxygen (Fig. 4b). CGRP in the lung tissue of premature rats reached its highest level after exposure to 95% oxygen for 7 days; at 14 days, the levels had declined but were still higher than in rats exposed to 21% oxygen for the same period (Fig. 4b).

### The expressions of Wnt7b, β-catenin, TCF and c-myc in AECII cells after CGRP exposure

**Fig. 5:**
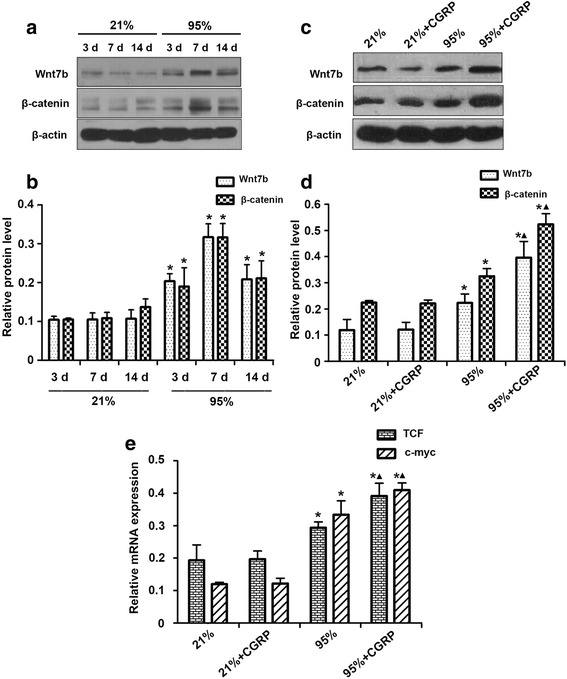
The expressions of Wnt7b, β-catenin, TCF and c-myc in AECII cells of premature rats exposed to 95% oxygen with CGRP exposure. **a** Protein expression of Wnt7b and β-catenin in the lung tissue of premature rats exposed to 21 or 95% oxygen, assessed via western blot analysis. **b** Relative protein levels of Wnt7b and β-catenin in lung tissues of premature rats exposed to 95% oxygen. *p < 0.05 vs. 21% group for the same period of exposure. **c** Protein expression of Wnt7b and β-catenin in AECII cells exposed to 95% oxygen with CGRP exposure. **d** Relative protein levels of Wnt7b and β-catenin in AECII cells. *p < 0.05 vs. 21% group; ^▲^*p* < 0.05 vs. 95% group. **e** Relative mRNA levels of TCF and c-myc in the lung tissues of premature rats exposed to 95% oxygen with CGRP exposure. *p < 0.05 vs. 21% group; ^▲^*p* < 0.05 vs. 95% group

To further understand the protective role of CGRP in oxidative injury, the expression levels of key components of the Wnt pathway were evaluated in AECII cells after CGRP exposure. Western blotting results demonstrated that the Wnt7b and β-catenin protein levels in the lung tissues of premature rats increased after 3 days of exposure to 95% oxygen. The Wnt7b and β-catenin protein levels in the lung tissues of premature rats reached the highest levels after 7 days of exposure to 95% oxygen and decreased after 14 days of exposure but remained higher than in premature rats exposed to 21% oxygen (Fig. 5a and b). The Wnt7b and β-catenin protein levels in AECII cells from premature rats exposed to 95% oxygen were significantly higher than in AEC II cells from rats exposed to 21% oxygen, and CGRP exposure further increased the Wnt7b and β-catenin protein levels (Fig. 5c and d). The qPCR results show that the TCF and c-myc mRNA levels in AECII cells from premature rats significantly increased after exposure to 95% oxygen compared the levels in the 21% group (Fig. 5e). CGRP exposure further increased TCF and c-myc mRNA levels in AECII cells (Fig. 5e).

## Discussion

We investigated the role of CGRP and the Wnt7b/β-catenin pathway in hyperoxia-induced lung injury in vivo and in vitro. Our results showed that exposure to 95% oxygen led to overproduction of MDA and upregulation of endogenous CGRP, resulting in oxidative lung injury in premature rats. We observed early congestion and infiltration of inflammatory cells, which led to destruction of the lung histopathology. CGRP_8–37_ exposure further abrogated the oxidant/antioxidant balance and aggravated the lung injury. In addition, oxidative lung injury was correlated with expression changes for Wnt7b and β-catenin.

Prolonged exposure to high concentrations of oxygen may cause oxidative injury to the lungs, as evidenced by previously reported significant ROS accumulation and increased expression of oxidative stress markers, including lipid peroxidation [25]. A large amount of free radicals and ROS can be produced by hyperoxia because the body is not able to scavenge O^2−^ [26]. The cell membrane contains a large amount of unsaturated fatty acids. Lipid peroxidation is initiated by the formation of potent oxidants such as OH^−^, and oxidation of unsaturated fatty acids produces monohydroperoxy derivatives (LOOHs) via interaction with O_2_ [27].

Here, we found that changes in the oxidative stress markers correlate significantly with lung injury, and hyperoxia exposure is associated with increased mortality rate and reduced body weight. Several studies have shown that exposure to hyperoxia significantly contributes to the pathogenesis of lung injury, and that lung injury consequent to prolonged hyperoxia is characterized by an overproduction of ROS, which could lead to an extensive inflammatory response [28].

CGRP, one of the major bioactive peptides secreted by the sensory nerves and neuroendocrine cells (PNECs), plays an important role in the regulation of airway reactivity and the function of airway epithelial stem cells, which are essential for lung development and injury repair [29, 30].

The major novel finding of this study is that the endogenous CGRP level increases in the lung tissues of hyperoxia-exposed rats, indicating that this may be an endogenous protective response. Some previous studies have shown that exposure to oxidants such as ozone increases the CGRP level in lung tissue [31] and that CGRP inhibits ozone-induced oxidative injury of airway epithelial cells [32], which is consistent with our results. It has also been reported that treatment with exogenous CGRP significantly alleviated hyperoxia-induced lung injury in neonatal rats [11]. These findings indicate that whether the CGRP is exogenous or endogenous, it could play a protective role in hyperoxia-induced lung injury.

We observed increased production of MDA and reduced production of TAOC in the lungs of premature rats exposed to high concentrations of oxygen, suggesting an oxidant/antioxidant imbalance. Prolonged exposure to high oxygen concentrations also caused alveolar structural remodeling, which was consistent with increased CGRP expression. These results suggest that CGRP is involved in oxidative injury to the lung and in alveolar remodeling. In addition, the CGRP antagonist, CGRP_8–37_, increased inflammation and further reduced the number of alveoli in the lungs of premature rats exposed to high oxygen concentrations, suggesting that the increased CGRP expression induced by exposure to high oxygen concentrations is an important protective mechanism in the premature lung to avoid oxidative injury.

In a previous study, it was reported that CGRP_8–37_ could enhance acute lung injury induced by lipopolysaccharide in a rat model and that it triggered an inflammation response [33]. In our study, when CGRP_8–37_ was used to inhibit the activity of CGRP in premature rats, the degree of lung injury was exacerbated by oxidative/anti-oxidative imbalance, which is consistent with the previous study [33]. Therefore, regulating endogenous CGRP may be a treatment for lung injury. The effect of an increase in CGRP observed is additionally supported by the results of a previous study, in which intravenously injected lipopolysaccharide resulted in increased endogenous CGRP in lung tissues from rats [34].

The changes in the Wnt7b/β-catenin signaling pathway molecules were investigated in AECII cells after exogenous CGRP exposure. We found that Wnt/β-catenin expression levels were elevated in the cells upon exposure to 95% oxygen, and that CGRP exposure further increased Wnt7b and β-catenin expression. The Wnt signaling pathway, including extracellular Wnt and intracellular β-catenin, plays an important role in the regulation of cell proliferation, differentiation and migration, and many transcription factors and cytokines are involved in its regulation [35]. Our results suggest that Wnt7b and β-catenin are involved in oxidative injury to the premature lung. Villar et al. reported that Wnt/β-catenin signaling pathway is activated in the progression of ventilator-induced lung injury [36], and it is known that inhibition of abnormal activated Wnt/β-catenin signaling would promote mesenchymal stem cell epithelial differentiation to repair lung injury and reduce pulmonary fibrosis [37]. Therefore, we hypothesized that the protective effect of CGRP in hyperoxia-induced lung injury may be associated with upregulation of endogenous CGRP and activation of Wnt7b/β-catenin signaling pathway.

High concentrations of oxygen increased the mRNA levels of TCF and c-myc in AEC II cells isolated from premature rats. Given that TCF is one of the most important downstream transcription factors in the Wnt pathway and that c-myc is a major target of TCF [38], our results suggest that high concentrations of oxygen activate c-myc through the Wnt7b/β-catenin pathway, which can promote AEC II cell proliferation.

## Conclusions

We found that exposure to high concentrations of oxygen caused congestion, inflammation and developmental alveolar stagnation in the lungs of premature SD rats. CGRP protected the lungs against oxidative injury induced by the high oxygen concentrations, and this was ultimately beneficial for lung development and oxidative injury repair. In addition, exposure to high oxygen concentrations altered the expression of Wnt7b and β-catenin in AEC II cells, resulting in the upregulation of TCF and c-myc, which demonstrated the involvement of the Wnt7b/β-catenin pathway in the protective role of CGRP against hyperoxia-induced lung injury.

### Limitations

Our results only demonstrated the involvement of Wnt7b and β-catenin in the protective role of CGRP against hyperoxia-induced lung injury. To investigate the molecular mechanism underlying the Wnt7b/β-catenin pathway in this situation, the inhibition or overexpression of Wnt7b/β-catenin should be assessed.

## Abbreviations

AEC II: Alveolar epithelial type II
BPD: Bronchopulmonary dysplasia
CGRP: Calcitonin gene-related peptide
MDA: Malondialdehyde
RAC: Radial alveolar counts
SPF: Specific pathogen free
TAOC: Total antioxidant capacity
